# Clinical Trials of Cannabidiol for Substance Use Disorders: Outcome Measures, Surrogate Endpoints, and Biomarkers

**DOI:** 10.3389/fpsyt.2021.565617

**Published:** 2021-02-22

**Authors:** Alix Morel, Pierre Lebard, Alexandra Dereux, Julien Azuar, Frank Questel, Frank Bellivier, Cynthia Marie-Claire, Mélina Fatséas, Florence Vorspan, Vanessa Bloch

**Affiliations:** ^1^Département de Psychiatrie et de Médecine Addictologique, Hôpital Lariboisière-Fernand Widal, GHU NORD, Assistance Publique – Hôpitaux de Paris, 200 rue du Fg St Denis, Paris, France; ^2^INSERM UMRS1144, 4 avenue de l'Observatoire, Paris, France; ^3^FHU NOR-SUD, Assistance Publique – Hôpitaux de Paris, Paris, France; ^4^UFR Médecine, Université de Paris, 3 rue Thomas Mann, Paris, France; ^5^University of Bordeaux, Bordeaux, France; ^6^CNRS-UMR 5287- Institut de Neurosciences Cognitives et Intégratives d'Aquitaine (INCIA), Bordeaux, France; ^7^Pôle d'addictologie, CHU de Bordeaux, Hôpital Haut-Lévêque, Avenue de Magellan, Pessac, France; ^8^Service de Pharmacie, Hôpital Fernand Widal, GHU NORD, Assistance Publique – Hôpitaux de Paris, 200 rue du Fg St Denis, Paris, France

**Keywords:** cannabis, tobacco, opioid, clinical trials, cannabinoids, cannabidiol, efficacy, biomarker

## Abstract

**Background:** Cannabidiol (CBD) is a cannabinoid of potential interest for the treatment of substance use disorders. Our aim was to review the outcome measures, surrogate endpoints, and biomarkers in published and ongoing randomized clinical trials.

**Methods:** We conducted a search in PubMed, Web of Science, PMC, PsycINFO, EMBASE, CENTRAL Cochrane Library, “clinicalTrials.gov,” “clinicaltrialsregister.eu,” and “anzctr.org.au” for published and ongoing studies. Inclusion criteria were randomized clinical trials (RCTs) examining the use of CBD alone or in association with other cannabinoids, in all substance use disorders. The included studies were analyzed in detail and their qualities assessed by a standardized tool (CONSORT 2010). A short description of excluded studies, consisting in controlled short-term or single administration in non-treatment-seeking drug users, is provided.

**Findings:** The screening retrieved 207 published studies, including only 3 RCTs in cannabis use disorder. Furthermore, 12 excluded studies in cannabis, tobacco, and opioid use disorders are described.

**Interpretation:** Primary outcomes were validated withdrawal symptoms scales and drug use reduction in the three RCTs. In the short-term or crossover studies, the outcome measures were visual analog scales for subjective states; self-rated scales for withdrawal, craving, anxiety, or psychotomimetic symptoms; and laboratory tasks of drug-induced craving, effort expenditure, attentional bias for substance, impulsivity, or anxiety to serve as surrogate endpoints for treatment efficacy. Of note, ongoing studies are now adding peripheral biomarkers of the endocannabinoid system status to predict treatment response.

**Conclusion:** The outcome measures and biomarkers assessed in the ongoing CBD trials for substance use disorders are improving.

## Introduction

The legalization of “medical marijuana” in several parts of the United States, soon followed by other countries, has produced an exponential increase in research using different active compounds derived from the *Cannabis sativa* plant in various medical conditions including substance use disorders (1).

Among those pharmacological agents, cannabidiol (CBD) may be the one provoking the highest expectations. For the general population, it is a painkiller and anxiolytic compound used either dermally as oil or orally as oil (2) or herbal tea, or smoked in electronic cigarettes (3, 4) for the self-treatment of several conditions associated with chronic pains, insomnia, and various psychological suffering. Compared with tetrahydrocannabinol (THC), CBD is the product of choice for medical cannabis users who do not have an associated recreational use (5).

Pharmacologically speaking, CBD is a CB1-receptor low-affinity agonist (6, 7) with inverse-agonist properties in the presence of THC (8). Targeting the specific CB1-receptor could be of interest in the treatment not only of cannabis use disorder. Indeed, it could be relevant also in depression, anxiety, or substance-related disorders in general for 3 reasons. First, this G-coupled protein is abundant and ubiquitous in the human brain, from the brainstem and cerebellum to the basal ganglia, hippocampus, and neocortex, thus regulating several important brain functions (9). Second, CB1 antagonists can provoke serious mood disorders (10), thus supporting the reverse hypothesis that CB1 agonists, including those with low affinity as CBD, might have antidepressant effects. Third, several genetic variants of *CNR1*, the CB1-coding gene, located on chromosome 6q14–15 (NC_000006.12), have been associated with either addictive (11–15) or mood disorders (16) in case–control studies, highlighting again the potential therapeutic properties of the pharmacological modulation of this target. The published GWAS of lifetime cannabis use and cannabis use disorders (17–19) did not confirm the association. However, the genetic risk conferred by minor alleles in *CNR1* is expected to have a small effect size and to interplay with several risk alleles for various psychiatric disorders. Still, genetic variants of *CNR1*, especially those located in the 3′UTR region, regulating the translation and stability of RNA, are good candidate biomarkers for treatment efficacy of pharmacological agents targeting the CB1-receptor.

Furthermore, CBD has several non-direct CB1-receptor effects, as demonstrated in animal or cellular models. It modulates the conformation of CB1- and CB2-receptor heteromeric complexes (8). It is also a strong agonist of TRPV (vanilloid channel receptors family) located on endothelial cells, including the blood–brain barrier (20), mediating its anti-inflammatory effects along with second messenger pathway activation. CBD inhibits the cellular reuptake of the endocannabinoid anandamide, increasing its activity (21) and also increasing its disposition (22). Lastly, CBD seems to have 5HT1-receptor agonist properties (23) and 5HT3a antagonist properties (24). Because of all those properties, CBD modulates dopamine, serotonin, opioid, and the brain inflammatory systems (25). CBD has shown several effects such as decreasing anxiety and depressive-like symptoms and decreasing pain and biological stress levels in several rodent models (26–28). As those symptoms are known triggers for relapse in substance use disorders (29, 30), those results from the pre-clinical literature suggest that CBD is an interesting candidate to test in human studies.

So far, CBD has demonstrated some anxiolytic properties in human studies (31), but most of this effect was obtained from studies where CBD was compared with THC, the major compound of smoked cannabis. CBD has also anticonvulsant properties (32), now well-established in controlled trials as an adjunctive treatment in child refractory conditions (Lennox–Gastaut and Dravet syndromes), and has a Food and Drug Administration (FDA) and European Medicines Evaluation Agency (EMEA) approval for those indications.

Concerning safety, in human studies, CBD has been safely administrated for several weeks to human subjects. CBD, especially, does not induce psychodysleptic effects or abuse. Indeed, as an add-on treatment of schizophrenia (33), at a dose of 1,000 mg per day during 6 weeks, CBD produced only a slight decrease in positive symptoms compared with placebo, but with acceptable tolerance (the main side effects being nausea in one-third of patients in the active group). CBD does not induce withdrawal symptoms as was shown by a specific trial assessing withdrawal symptoms after 4 weeks of CBD 750 mg twice a day and either blind maintenance or abrupt cessation under placebo (34). In this trial, as in the literature, to the best of our knowledge, no study described any case of CBD use disorder.

Concerning efficacy, CBD has shown some promising properties in pre-clinical studies and some clinical studies in the field of psychiatry and addiction medicine. To help identify the methods currently used to assess the potential therapeutic properties of CBD in substance use disorders and isolate them from the noise of high expectations, we choose to perform a review of both published and ongoing randomized clinical trials in humans. We present the studies with a specific focus on the outcome measures, surrogate endpoints, and biomarkers developed by the authors to show clinical efficacy or at least to show that CBD could modify targets associated with efficacy in substance use disorders.

## Methods

### Search Strategy and Selection Criteria

First, we conducted a review of the published clinical trials through a PubMed data search. Looking for double-blind randomized trials, published before May 2020, we led 10 separate searches. CBD could be assessed alone or in association with other cannabinoids, in (a) alcohol, (b) amphetamine, (c) cannabis, (d) cocaine, (e) hallucinogen, (f) inhalant, (g) opioid, (h) phencyclidine, (i) sedative, and (j) tobacco use disorder.

We used the following terms: “(cannabidiol OR CBD) AND (randomized trial OR randomized study) AND (substance related disorder OR addiction OR use disorder OR use OR abuse OR excessive use OR dependence OR withdrawal)” AND either “(alcohol),” “(amphetamine OR speed OR stimulant),” “(cannabis OR marijuana OR THC),” (cocaine OR crack OR freebase),” “(hallucinogen),” “(inhalant),” “(opioid OR heroin),” “(PCP OR phencyclidine OR angel dust),” “(benzodiazepine OR sedative),” or “(tobacco OR nicotine).” Inclusion criteria for the articles were double-blinded, randomized, placebo, or adequate control, in subjects with a formal diagnosis of substance use disorder, assessing CBD alone or in association with other cannabinoids, and reporting at least one primary outcome regarding substance use disorder.

Exclusion criteria were as follows: studies involving healthy volunteers, single administration, pre-clinical studies, reviews, opinion papers, protocols, open-label studies, case reports, and studies not published in English.

Two authors (AM and PL) independently examined titles and abstracts. Relevant articles were obtained in full text and assessed for inclusion criteria blindly by the two reviewers. Disagreement was resolved *via* discussion to reach consensus.

Detailed data on each included randomized controlled trial, including target population, intervention, treatment dose, frequency and route of administration, treatment duration, control group, outcome measures, surrogate endpoints and biomarkers, adverse events, and study withdrawals, are described. The risk of bias was assessed with the Cochrane risk of bias tool, which includes assessment of indicators of selection bias, performance bias, detection bias, attrition bias, and reporting bias. Furthermore, the CONSORT 2010 (Consolidated Standards of Reporting Trials) statement was used to rate the report made in each article of the study design, analysis, and interpretation.

For the excluded studies consisting in short-term or single-administration, proof-of-concept studies, conducted mostly in non-treatment-seeking drug users, only a shorter presentation of outcome measures, surrogate endpoints, and biomarkers is provided.

Secondly, to ensure that no RCT was missed, we conducted another search with the same key words in Web of Science, PMC, PsycINFO, EMBASE, and CENTRAL Cochrane Library. No further study was added.

Lastly, to identify ongoing or unpublished studies, we searched different online registries: “clinicaltrials.gov,” “clinicaltrialsregister.eu,” and “anzctr.org.au” websites, using the terms “substance related disorder OR addiction OR use disorder OR use OR abuse OR excessive use OR dependence OR withdrawal” and “cannabidiol OR CBD OR nabiximols OR (THC + CBD).”

## Results

**Figure 1:**
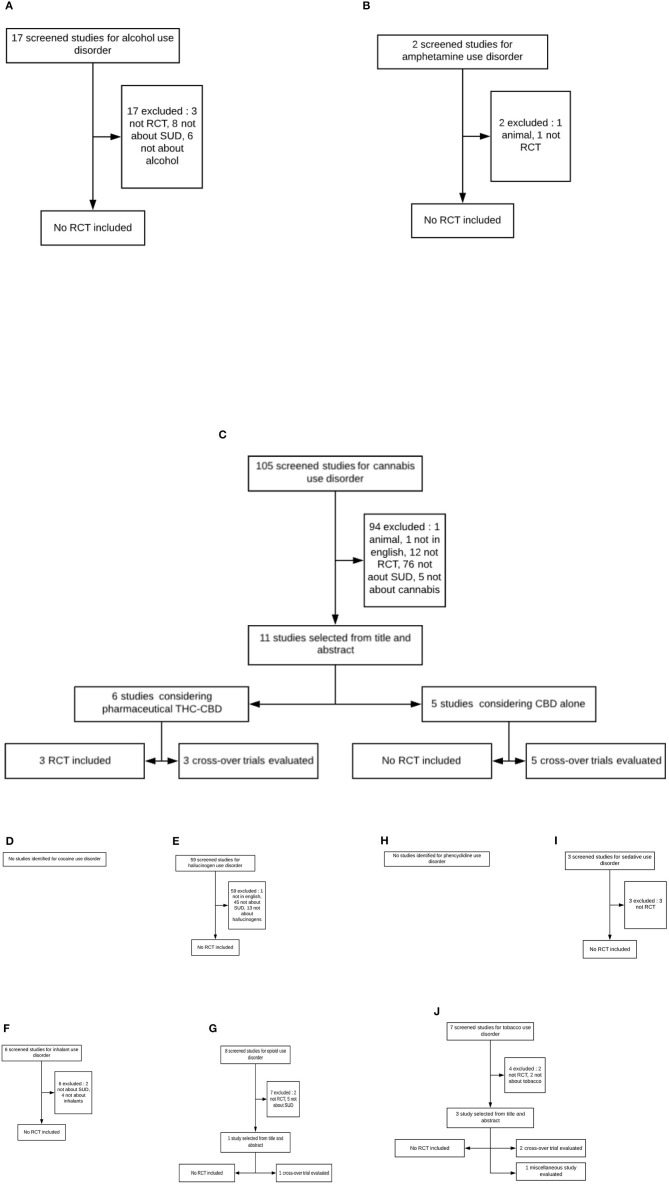
Flowchart of screened, selected, included, and evaluated studies (SUD, substance use disorder; RCT, randomized controlled trial). **(A)** Alcohol use disorder. **(B)** Amphetamine use disorder. **(C)** Cannabis use disorder. **(D)** Cocaine use disorder. **(E)** Hallucinogen use disorder. **(F)** Inhalant use disorder. **(G)** Opioid use disorder. **(H)** Phencyclidine use disorder. **(I)** Sedative use disorder. **(J)** Tobacco use disorder.

The PRISMA flowcharts presenting the selection of studies are shown in Figure 1. The initial screening identified 17 published articles presenting studies assessing CBD for alcohol, 2 for amphetamine, 105 for cannabis, 59 for hallucinogen, 6 for inhalant, 8 for opioid, 3 for sedative, and 7 for tobacco use disorder. All the other researches that we conducted retrieved no results. Of those screened studies, we finally retained only 3 studies meeting the inclusion criteria with a classical design of randomization in parallel groups, vs. placebo, for cannabis use disorder, all assessing the efficacy of nabiximol spray (a 1:1 THC/CBD ratio). Their outcome measures, surrogate endpoints, and biomarkers are detailed in Table 1.

**Table 1:** Characteristics of the 3 included randomized controlled trials assessing inhaled tetrahydrocannabinol–cannabidiol (THC–CBD) in cannabis use disorder.

**Author**	**Allsop et al. (35), Australia**	**Trigo et al. (36), Canada**	**Lintzeris et al. (37), Australia**
Number of subjects	P, *n* = 24/N, *n* = 27	P, *n* = 20/N, *n* = 20	P, *n* = 73/N, *n* = 64
Out-/inpatient	Inpatient	Outpatient	Outpatient
Withdrawal	During withdrawal	During withdrawal/follow-up	During withdrawal/follow-up
Treatment	Self-titrated Maximum 86.4 mg THC + 80 mg CBD/day + CBT	Self-titrated Maximum 113.4 mg THC + 105 mg CBD/day + MET/CBT	Self-titrated Maximum 86.4 mg THC + 80 mg CBD/day + CBT
Duration	6 days of treatment, 3 days of washout, 28 days of follow-up	12 weeks	12 weeks
Primary outcome	(Intervention) Withdrawal score	Cannabis use, tolerability	Cannabis use
Secondary outcome	(Intervention) Craving (Follow-up) Time to relapse Use reduction Psychosocial outcome Tolerability	Craving score, withdrawal score	Abstinence, use reduction, withdrawal score, craving score, tolerability
Outcome measures	CWS Urine and plasma drug test	TLFB (7 days) Urine and plasma drug tests MWC MCQ-SF	TLFB (28 days) Urine drug test (placebo group) MWC MCQ
Main results	CWS: N (−66%) > P (+52%), *p* = 0.01 Retention: N > P at day 6	Cannabis use: NSD Tolerability: NSD	P (53/84 d) > N (35/84 d), *p* = 0.02
Secondary results	Time to relapse: NSD Reduction use: NSD Psychosocial: NSD Tolerability: NSD	Withdrawal: NSD Craving: NSD	Abstinence: NSD Withdrawal: NSD Craving: NSD
Quality	CONSORT: 31/32 Biases 1/10	CONSORT: 24/32 Biases 2/10	CONSORT: 30/32 Biases 3/10

*MET/CBT, motivational enhancement therapy and cognitive behavior therapy; CBT, cognitive behavior therapy; TLFB, Timeline Followback; MWC, Marijuana Withdrawal Checklist; MCQ, Marijuana Craving Questionnaire; MCQ-SF, Marijuana Craving Questionnaire Short Form; CWS, Cannabis Withdrawal Scale; NSD, non-significant difference; P, placebo; N, nabiximol*.

Although not properly speaking randomized controlled trials of efficacy, 12 other controlled studies are presented: 3 studies of THC–CBD combination on various endpoints in cannabis users and 9 studies assessing the efficacy of CBD alone, mostly as oral tablets, on surrogate endpoints of efficacy for cannabis (4 studies), opioid (1 study), tobacco dependence (3 studies), or multiple substance use (1 study). The main considerations for exclusion are detailed in Figure 1.

### Outcome Measures, Surrogate Endpoints, and Biomarkers of the Three Randomized Controlled Trials Assessing THC–CBD in Cannabis Use Disorder

#### Withdrawal Symptoms

Only three trials randomized by group, as well as placebo-controlled, assessed the pharmaceutical preparation nabiximol (1:1 THC/CBD ratio) for cannabis use disorders (30–32). All 3 studies took place in subjects with verified cannabis use disorder criteria during a cessation attempt. The studies lasted between 6 days and 12 weeks, giving way to observe both early withdrawal symptoms and later abstinence maintenance or relapse, but also to quantify cannabis use. The first published study (35), conducted in 6 consecutive days in hospitalized patients, chose to assess the CWS (Cannabis Withdrawal Scale) (38), a self-rated withdrawal scale, as the main outcome.

#### Drug Use Reduction

In the two other RCTs, the investigators assessed 12-week cannabis use reduction with self-reports collected with the Timeline Followback as their primary outcome (see Table 1) and relegate withdrawal symptoms questionnaires as secondary outcome measures. Of note, in those studies, abstinence, defined as a 4-week cannabis cessation, and time-to-relapse were also only secondary outcomes. Furthermore, the 3 studies added urine or plasma cannabis measurement to characterize drug use reduction and act as surrogate endpoint predictors of abstinence. The 3 trials included a validated self-rated craving questionnaire, the Marijuana Craving Questionnaire (MCQ) (39), either complete or short form, as surrogate endpoints for abstinence. None of those 3 studies used biomarkers as a potential predictor of abstinence or cannabis use reduction.

### Quality of the Methodology of the Randomized Controlled Trials

Overall, the quality of those 3 studies was good. The detailed risk of bias and quality rating regarding those studies are presented in Tables 2, 3. Analyses were performed in intention-to-treat and missing data were handled by several appropriate methods: multiple imputation (35), maximum likelihood estimation (36), or intention-to-treat restricted to subjects who had received at least one dose of medication (37).

**Table 2:** Internal and external validity of the 3 THC-CBD trials in cannabis use disorder evaluated by Cochrane risk of bias tool.

	**Allsop et al. (35)**	**Trigo et al. (36)**	**Lintzeris et al. (37)**
**Internal validity**			
**Selection bias**			
Random sequence generation	y	y	y
Allocation protected from contamination	y	y	y
Similar baseline characteristics	y	y	y
**Detection bias**			
PPG calculation	y	y	y
Blinding of outcome assessment	y	y	y
Adequate outcome measurement	y	y	y
Equivalent assessment	y	y	y
**Attrition bias**			
Incomplete outcome data	y (ITT)	y (ITT)	y (mITT)
**Report bias**			
No selective reporting	y	y	y
**External validity**			
Appropriate comparator	y	y	y

*PPG, participant per group; y, yes; ITT, intention-to-treat; mITT, modified intention-to-treat*.

**Table 3:** CONSORT quality ratings of the 3 THC-CBD trials in cannabis use disorder.

**CONSORT 2010**			**Allsop et al. (35)**	**Trigo et al. (36)**	**Lintzeris et al. (37)**
Title and abstract		1a	y	n	y
		1b	y	y	y
Introduction		2a	y	y	y
		2b	y	y	y
Methods	Trial design	3a	y	y	y
		3b	n/a	n/a	n/a
	Participants	4a	y	y	y
		4b	y	n	y
	Interventions	5	y	y	y
	Outcomes	6a	y	n	y
		6b	n/a	n/a	n/a
	Sample size	7a	y	y	y
		7b	n/a	n/a	n/a
Randomization	Sequence generation	8a	y	y	y
		8b	y	y	y
	Allocation concealment mechanism	9	y	n	n
	Implementation	10	n	y	y
	Blinding	11a	y	y	y
		11b	y	y	y
	Statistical methods	12a	y	y	y
		12b	y	y	y
Results	Participant flow	13a	y	n	y
		13b	y	y	y
	Recruitment	14a	y	n	y
		14b	n/a	n/a	n/a
	Baseline data	15	y	y	y
	Numbers analyzed	16	y	n	y
	Outcome measures and estimation	17a	y	y	y
		17b	n/a	n/a	n/a
	Ancillary analyses	18	y	y	y
	Harms	19	y	y	y
Discussion	Limitations	20	y	y	y
	Generalizability	21	y	y	y
	Interpretation	22	y	y	y
Other information	Registration	23	y	y	y
	Protocol	24	n	n	y
	Funding	25	y	y	y
Total/32			31	24	30

*n, no; y, yes; n/a, non-applicable*.

### Outcome Measures, Surrogate Endpoints, and Biomarkers of the 12 Excluded Studies

Here, we give a short presentation of the methodology of the 12 pilot controlled studies that are not RCTs enrolling treatment-seeking subjects.

#### Three Crossover Trials Assessing THC–CBD in Cannabis Use Disorders

##### Consecutive Administration

*Withdrawal Symptoms* The study by Trigo et al. (40) used a crossover design in 16 participants with cannabis use disorder to assess withdrawal symptoms during repetitive 5-day cannabis cessation sessions assessing several doses of nabiximol. The primary outcome was assessed by 2 withdrawal scales: the CWS (38) and the Marijuana Withdrawal Scale (MWC) (41). A validated self-rated craving score, the MCQ (39), was used as a secondary outcome measure, as were the side effects or the quotation of feeling “high” with the THC–CBD doses.

*Single Administrations* Two crossover controlled studies assessing the effect of a single administration of THC–CBD or CBD alone used motivation and anxiety measures as primary endpoints.

*Motivation and Reward Expectation* One study chose to assess the motivation for rewarded tasks as a primary outcome measure (42). In a double-blinded placebo-controlled experimental study, 17 subjects realized an effort expenditure for rewarded tasks, under 3 conditions: after THC or THC–CBD (vaporized 8 mg THC + 10 mg CBD) or placebo inhalation. The authors measured not only the amount of the effort produced but also the amount of expected reward associated with the effort produced. The authors observed that CBD could attenuate the indifference provoked by THC, expressed in the attenuation of expected reward.

*Anxiety* Another study (43) reported more classical outcome measures in terms of heart rate and blood pressure and several self-rated visual analog of mood states including good drug effect and high anxiety, but also the repetitive assessment state anxiety part of the Spielberger State–Trait Anxiety Inventory (38). Those assessments were repeated several times over 10 h after a single intake of either the following: oral THC 5 mg, oral THC 15 mg, oromucosal spray pharmaceutical THC–CBD low dose (5.4 mg THC + 5.0 mg CBD) or high dose (16.2 mg THC + 15.0 mg CBD), oral placebo, or oromucosal spray placebo. The subjects were 9 occasional cannabis users. The adjunction of CBD did not prevent the rise of anxiety associated with THC in the few hours after THC–CBD mixtures.

### Outcome Measures, Surrogate Endpoints, and Biomarkers of the Nine Excluded Studies of CBD Alone for Substance Use Disorders

#### Consecutive Administration

##### Drug Use Reduction

We identified a pilot study in tobacco dependence (44) with only an indirect comparison design that did not qualify for our inclusion criteria. We thus classified it as “miscellaneous” (see Figure 1J). The chosen primary outcome was smoking reduction measured by the declared number of cigarettes smoked in 1 week. Smokers were randomized to receive either *ad libitum* inhaled CBD (*n* = 12) or placebo (*n* = 12) *via* an inhaler delivering 400 μg of CBD at each press. Secondary outcome measures included tobacco craving and self-rated separate visual analog scales of the MRS (Mood Rating Scale) (45) including depression, anxiety, and sedation. The results are presented like those assessments that occurred only once on day 0 and once on day 7. No direct comparison of craving reduction between groups is provided.

#### Single Administrations

We present here some data from the eight other published articles of interest. They were conducted in heroin-dependent subjects (1 study), in regular cannabis users (4 studies), in dependent tobacco smokers (2 articles), and in subjects with multiple dependencies (1 study). Their primary outcome measures were diverse and are listed below.

##### Cue-Induced Craving and Anxiety

The only published study assessing CBD effects in 42 subjects with heroin use disorder, currently abstinent (46), was a crossover, placebo-controlled trial examining 3 consecutive days of oral CBD 400 mg per day or CBD 800 mg per day or placebo. The primary outcome measures were repetitive visual analog scales (VASs) of craving and anxiety during cue-induced laboratory sessions, up to 7 days after the end of CBD administration. Several secondary outcome measures were also assessed: the Positive and Negative Affect Scores (PANAS) (47) and several cognition tests, mostly consisting in sustained attention tasks, such as a Digit Symbol Substitution Task (DSST), a Digit Span Test–Backward (DSTB), and a Continuous Performance Task (CPT). The investigators added physiological measures, including heart rate, blood pressure, and body temperature and salivary cortisol levels, as biomarkers of cue-induced stress during the exposition task. The authors concluded that both CBD doses reduced craving and anxiety during the tasks of salient drug cue presentation compared with neutral cues. In addition, the drug cue-induced physiological measures of heart rate and salivary cortisol levels were also attenuated. No sedation effects were observed, and there was also no cognitive enhancement.

##### Psychomimetic Subjective Effect

A study conducted in occasional and regular cannabis users with a single inhalation of either THC 8 mg, CBD 16 mg, THC 8 mg + CBD 16 mg, or placebo (48) chose as primary endpoint a scale designed to assess drug-induced psychotomimetic effects, the Psychotomimetic States Inventory (PSI) (49) along with the validated Brief Psychiatric Rating scale (BPRS) (50). The co-administration of CBD did not attenuate the psychotomimetic effects of THC, and CBD alone reduced PSI scores in light users only. This study included a working memory task using a word list and sustained attention tests as secondary outcome measures, showing again that CBD in co-administration did not attenuate the impairing memory and cognitive effect of THC and that CBD alone had no cognitive enhancement properties.

##### Attentional Bias and Impulsivity

In order to test what could be surrogate endpoints for CBD efficacy in tobacco use disorder, a British team published in 2 interesting articles the results of a crossover trial of 1 administration of 800 mg CBD vs. placebo in non-treatment-seeking tobacco smokers during experimental sessions of 24 h abstinence, separated by 1-week washouts (51, 52). In the first report (51), the primary outcome was the attentional bias toward tobacco cues (AB) as a slower response time during a Visual Probe Task (VPT) with both neutral and smoking-related cues. Furthermore, participants had to quote the pleasantness of the task. Secondary outcome measures included withdrawal and craving scales, heart rate, blood pressure, and side effect scales. In the second report (52), the primary outcome was impulsivity, and it was measured by 2 tests. In a Delay-Discounting Task, no significant difference between CBD and placebo was found, while a Go/No-go task showed significantly more errors with CBD than placebo. Memory was measured by a Prose Recall Task (PRT), showing no significant difference between CBD and placebo. Furthermore, an N-Back Task (NBT) showed no difference for correct responses, reaction time, and maintenance and manipulation. Thus, CBD was not shown to improve cognition in the specific condition of nicotine withdrawal.

##### Cognitive Performance

Several cognitive tests were also assessed in another specific condition, this time the pretreatment with a single dose of 200, 400, or 800 mg CBD prior to smoked cannabis intake (53), along with several VASs exploring the reinforcing and subjective effect of this interaction, during 8 sessions. Once again, no specific significant differences were found between CBD and placebo and neither was there any signal of abuse liability (54).

##### Abuse Liability

Another team performed the same kind of experiment to assess the abuse liability of oral CBD in healthy recreational polydrug users (55). The investigators compared single administrations of 750, 1,500, and 4,500 mg oral CBD to alprazolam 2 mg (APZ) or dronabinol (THC) 10 and 30 mg. The primary outcome was again the maximum effect (Emax) on a drug-liking VAS scale, with also positive (“feeling high” and “feeling stoned”) and negative effects, and there were several other subjective effects as secondary outcome measures. Cognitive, memory, and psychomotor functions were measured by a Divided Attention Test (DAT), the Hopkins Verbal Learning Test Revised (HVLT), and the DSST. Again, this study confirmed that single-dose oral CBD does not show any signal of abuse liability as well as no detectable cognitive effect in this condition.

##### Facial Emotion Recognition Task

Originally, we identified 1 study conducted by Hindocha et al. (56), which examined the acute effects of THC, CBD, and their combination on facial emotion recognition. This task consists in showing six basic emotions (happiness, sadness, anger, disgust, fearful, surprise, and neutrality) and with an intensity degree in 5 levels. Facial recognition is impaired in mood and anxiety disorders. The reduction of its impairment is proposed as a surrogate endpoint for treatment efficacy in anxiety disorders when screening new molecules (48). Regular cannabis smokers attended 4 sessions with a 1-week washout and were administered by inhalation either THC 8 mg, CBD 16 mg, THC + CBD (8 + 16 mg), or placebo. The results showed that at 60% intensity, participants were more accurate with CBD alone than placebo. At more ambiguous emotion levels, at 40% intensity, participants with THC–CBD were more accurate than participants with THC alone. As a secondary outcome measures, participants also completed the subjective effect VASs for “stoned,” “anxiety,” “alert,” and “happy or sad,” among other subjective states. The results did not support the investigators' hypothesis that cannabis users would differ according to their score on a Schizotypal Proneness Questionnaire.

None of those single-administration studies tested if their primary or secondary outcome measures were associated with indirect brain biomarkers of substance use disorder severity or evolution. In the studies presenting time-curve evolution of mood or cognitive effects over some hours, no correlation with plasma CBD level was shown.

#### Ongoing Studies

Our screening in the American clinical trial registry (clinicaltrials.gov) identified 87 studies. The same screening in the European clinical trial web-base (clinialtrialsregister.eu) identified 2 studies and seven from the Australian and New Zealand clinical trial registry (anzctr.org.au). We did not retain studies not performed in substance use disorders (mostly performed in epilepsy or chronic pain), already published studies (previously included in this review), and studies recorded in several registries. This left 13 studies. Of note, and this is an important change from the past few years, all those studies are evaluating the efficacy of CBD alone as the treatment of interest. The substance use disorder conditions assessed in those studies were as follows: cannabis use disorder (UD) (5 studies), opioid UD (4 studies), alcohol UD (3 studies), and 1 study was also found in cocaine UD. Eight studies were conducted in North America, 2 in Europe, 2 in Oceania, and 1 with unknown location. Protocols, CBD dose, and duration vary according to the study. The duration of CBD administration ranges from four single administrations to 3 months, with the majority of studies assessing 1–2 months of treatment. CBD doses range from 300 to 1,400 mg per day. The primary outcome measures are withdrawal symptoms or craving in the shortest studies (on opioid UD, alcohol UD) and also substance use or relapse, associated with craving in several-week duration trials (cannabis UD, alcohol UD, cocaine UD). Most studies have also secondary outcome measures with various subjective symptoms scales: anxiety, sleep quality, psychotic symptoms, and craving, serving as surrogate endpoints for efficacy. On top of that, those more recent studies add several biomarkers to be tested as surrogate endpoints for efficacy: cannabidiol plasma levels (alcohol UD studies, opioid UD studies, cocaine UD study), combined with endocannabinoid plasma levels (cannabis UD studies and cocaine UD study) composed of both CBD and anandamide plasma levels, sometimes combined with other biomarkers: mono-amine plasma levels or inflammatory biomarkers including plasma cortisol in cocaine UD.

## Discussion

Despite the great expectations toward the possible therapeutic effects of CBD in substance use disorders, this review showed that published data are limited. There is no published study demonstrating the efficacy of CBD alone to treat any substance use disorder.

When choosing stringent inclusion criteria, only 3 high-quality randomized placebo-controlled trials can be retained. All those 3 studies tested THC/CBD compounds and proposed to treat cannabis use disorder. Their primary outcome measures were validated scales of withdrawal symptoms or cannabis use reduction. In the context of efficacy trials, validated craving scales, previously associated with relapse, are only secondary outcomes. Those 3 studies did not report on any biomarker that could be used as a useful predictor of efficacy.

Regarding trials assessing CBD alone in treating substance use disorder, none of them can qualify as a high-quality randomized controlled trial. Published data are limited to very short-term or even single-administration crossover designs. In such short-term pilot studies, the efficacy assessment can only rely on primary outcome measures sensitive to short-term change. In that context, series of visual analog scales of various subjective effects, describing the drug effects or anxiety or mood states, are useful and allow repetitive assessments and the establishment of time curves. The adjunction of validated withdrawal or craving scales, as well as scales assessing anxiety or psychotomimetic effects, is an improvement if those scales are validated for such repetitive assessments.

The investigators identified tasks that could be surrogate endpoints for treatment efficacy in substance use disorders, by mimicking conditions associated with relapse: drug-induced craving, attentional bias for the substance, impulsivity, or anxiety. The assessment of the expected procognitive properties of CBD does not target relapse. It is rather a way to rule out the THC-induced cognitive side effects.

There is a shift in the most recently declared clinical trials toward more prolonged efficacy trials and toward targeting more substance use disorders, including alcohol and cocaine use disorders. This shift is also accompanied by a qualitative improvement of the methodology toward the use of biomarkers that could be predictive of CBD efficacy. Above classical pharmacokinetic parameters such as CBD plasma level, which could help to define a therapeutic range, researchers are now adding new peripheral biomarkers assessing the current state of the endocannabinoid system, the mono-amine system, or the immune system. Of note, those biomarkers could be applied to all substance use disorders. Indeed, repetitive drug intake produces homeostatic changes in the common final pathway of the brain reward circuit. The endocannabinoid system plays a role of modulator of this circuit. Those therapeutic trials could benefit from a more general enhancement in research for the identification of valid biomarkers of the reward circuit homeostatic state. They could include peripheral biomarkers, combined with brain imagery or neuropsychological tasks, and eventually drug administration challenges to describe the various stages of substance use disorder. In particular, an entire research era consisting in the design of study protocols able to assess the central nervous system pharmacological target engagement by CBD could emerge in the next years. They could include the association of *CNR1* gene polymorphisms with treatment response, or specific measures of the central nervous system inflammation state through radioactive ligands, or markers of CB1- or 5HT-receptors or TRPV channel activity.

Among the strengths of our review, we would like to point out the stringent definition of included/excluded published studies; the extended search strategy including PubMed, Web of Science, PMC, PsycINFO, EMBASE, and CENTRAL Cochrane Library; the double selection made independently by two reviewers; and the separate presentation of declared ongoing studies.

## Conclusion

The field of research assessing the efficacy of CBD in substance use disorder is emergent. To date, published randomized controlled trials are limited to THC–CBD compounds. However, pilot studies assessing single administrations or short-term efficacy of CBD alone on surrogate endpoints of efficacy have already been conducted. They targeted cue-induced craving, effort expenditure, attentional bias for the substance, impulsivity, or anxiety. The next generation of trials, already ongoing, will include peripheral biomarkers of the endocannabinoid system homeostatic state as well as immunologic biomarkers as potential predictors of efficacy. Our recommendation for future randomized clinical trials testing the efficacy of CBD to treat substance use disorders would be to combine the repetitive assessment of 3 types of biomarkers of efficacy: peripheral biomarkers of the endocannabinoid system such as cannabinoid plasma level, short-term surrogate endpoints (such as craving or attentional bias reduction), and long-term validated measures of abstinence, dose reduction, or harm reduction.

## Author Contributions

FV and MF designed the study. AM and PL screened the studies and wrote the first draft. All authors have significantly contributed to the discussion and approved the final manuscript.

## Conflict of Interest

The authors declare that the research was conducted in the absence of any commercial or financial relationships that could be construed as a potential conflict of interest.
